# On the Acidity and Alkalinity of Certain of the Human Fluids in the State of Health and Disease

**Published:** 1848-11

**Authors:** 


*On the Acidity and Alkalinity of certain of the Human Fluids in the state of Health and Disease.* By M. Andral.—In their physiological conditions, each of the humours of the body presents a certain degree of acidity or alkalinity; and the spotaneous transformation of a naturally acid fluid into an alkaline one, or *vice versâ*, never takes place in the healthy organism. The utmost that can occur in this respect, is the rendering the fluid temporarily neutral by great dilution, as in the case of excessive perspiration—the water being then abstracted from the blood in larger proportion than the other principles. However this may be in health, the opinion is very generally entertained that in disease such change in the humours does often take place; and the object of this paper is to investigate its accuracy.

Of all the fluids of the economy, the *serum of the blood* is the most decidedly alkaline; and whatever the nature of the disease or its duration, in which M. Andral has examined this fluid, he has never found the intensity of this reaction sensibly vary. Vogel quotes a case of metro-peritonitis from Scherer, in which the serum of the blood was said to be perfectly neutral, but adds, that he himself had never met with any thing similar. If blood is examined after death, any acidity then found is the result of decomposition, and not the effect of disease. In examining the condition of the fluids formed from the blood, it should be borne in mind that upon the same surfaces liquids possessed of different reactions may be found; so that the accidental predominance of one of these fluids may easily be mistaken for a change in the reaction of another. Thus the sweat is acid, but the sebaceous matter is alkaline. In the very various conditions in health and disease under which M. Andral has examined the sweat, he has found it generally acid, sometimes from dilution neutral, never alkaline; but at the same time, at some parts of the skin, where sebaceous follicles abound, as the axilla and other hairy parts, an alkaline reaction may exist. It is evident, then, that the sweat is not a simple escape of the serum of the blood, charged with certain of its principles, for then it would be alkaline; and if the skin be irritated by blisters and the like, the fluid consequently effused will be found decidedly alkaline. So is the fluid found in herpes, eczema, pemphigus, &c., vesicular diseases preceded by more or less congestion of the skin; and it remarkable that the contents of sudamina, which unlike these are preceded by no congestion, are acid, being also destitute of albumen, which is found in the others. Although sudamina are usually accompanied by excessive sweating, cases of typhoid fever are sometimes met with where this is not the case.

Still more remarkable is the difference of the reactions of the various fluids found on mucous membranes, giving rise to considerable chance of error. Throughout their whole extent, they furnish, like the skin, an acid principle, which exists in the transparent fluid, destitute of globules, which they normally separate from the blood; but when this fluid is replaced by one of an opaque appearance and containing globules, secreted under the influence of acute or chronic inflammation, the reaction becomes decidedly alkaline. Few animal fluids are so strongly alkaline as that furnished in coryza; and in bronchitis the acid and alkaline are not unfrequentlyfound together, and yet remaining quite distinct, in their transparent and opaque forms. The mucous membrane of the mouth and tongue, too, offers varieties of condition. Examined in the morning, before food is taken, in the vast majority of cases, the fluid covering it is acid; but examined later in the day this is found to be alkaline. In the first case, it is due to the presence of the mucus; in the latter to that of the saliva. The acidity of the mouth is then no indication of a morbid condition of the stomach, occurring as it does in the healthiest persons, and in every variety of disease, and being distinct in proportion to the length of time food has been abstained from, and the secretion of the salivary glands has remained unexcited. The mucous membrane of the stomach, examined after death, generally furnishes an acid, sometimes a neutral, but never an alkaline reaction; and this whether it yet contains the remains of food, or whether digestion has been long suspended. How are we to reconcile this with the results of experiments which declare the fluids of the organ to be alkaline, save when stimulated by the presence of food or foreign bodies? This is not the case in man; for in the most opposite forms of disease the author has found acidity; and the great majority of matters rejected during life manifest the acid reaction. It is not rare to find this also in the duodenum and upper portions of small intestine; although these are often rendered alkaline by the arrival of the fluids from the liver and pancreas. Throughout the large intestine there is always marked alkalinity. The tears and saliva have always been found by M. Andral alkaline; and he believes that when this latter fluid has been said to be otherwise, that of the mucous membrane has been mistaken for it. Thus in the very cases furnishing an acid reaction, if we, by means of a sapid body, excite the flow of the saliva, we immediately find this alkaline. "And thus falls to the ground one of the principal arguments which has been adduced in support of the theory which regards glucosuria as resulting from the acidification of the blood or other humours of the economy."

In a state of health, urine which has not remained too long in the bladder, and is examined soon after voiding, is always acid, although such acidity may become enfeebled, or even neutralized, if very abundant drinks be taken without corresponding diaphoresis. Circumstances may render the urine temporarily alkaline, as the taking alkalies, or the prolonged use of exclusively herbivorous aliment. The privation of food, however long, does not remove the acidity of the urine; but it is a curious fact, that in some convalescents we find the urine become temporarily alkaline when they commence a better diet. Nor does disease render the urine alkaline. Multiplied as have been the author's observations upon this point, he has never met with a case in which the urine, from the influence of the disease itself, left the kidneys in an alkaline state; and he feels convinced that the statements which have been to the contrary made are founded in error. It has been said that diseases of the spinal marrow have this effect; but, in fact, the urine never becomes alkaline in these, until the mucous membrane of the bladder is diseased. It is not then an alteration of secretion, but a purely chemical one; the urine becoming decomposed and ammoniacal, from coming in contact with pus and other morbid products. Pus, whatever its source, is always alkaline, consisting as it does of the serum of the blood, amidst which special globules are developed ; and this, as well as other morbid secretions, never becomes acid, except after long exposure to the air.

The immutability of the secretion of the acid and alkaline principles of the animal fluids is then a law of both their physiological and pathological conditions ; and it must be a very important one, seeing that it persists without any exception, save one of a very temporary character, in respect to the influence of alimentary substances in the urine.—*Gazette Médicale.*

